# Enhanced Electrocatalysts for Oxygen Reduction Reaction: Insights from Accelerated Stress Testing and IL-TEM Analysis

**DOI:** 10.3390/nano15100776

**Published:** 2025-05-21

**Authors:** Angelina S. Pavlets, Elizaveta A. Moguchikh, Ilya V. Pankov, Yana V. Astravukh, Sergey V. Belenov, Anastasia A. Alekseenko

**Affiliations:** 1 Faculty of Chemistry, Southern Federal University, 7 Zorge St., Rostov-on-Don 344090, Russia; pavlec@sfedu.ru (A.S.P.); moguchih@sfedu.ru (E.A.M.); astravukh@sfedu.ru (Y.V.A.); serg1986chem@mail.ru (S.V.B.); 2 Research Institute of Physical Organic Chemistry, Southern Federal University, 194/2 Stachki St., Rostov-on-Don 344090, Russia; ipankov@sfedu.ru

**Keywords:** electrocatalysts, PtCu nanoparticles, de-alloyed structure, nitrogen-doped carbon, identical location transmission electron microscopy, accelerated stress test, ORR

## Abstract

This report introduces a high-performance bimetallic electrocatalyst for the oxygen reduction reaction (ORR) featuring a 20 wt.% platinum content. The PtCu-based catalyst combines de-alloyed nanoparticles (NPs) supported on nitrogen-doped carbon. Enhanced uniformity in NP distribution significantly boosts the catalyst performance. Nitrogen-doped carbon provides active centers for NP deposition, which is confirmed by HAADF-STEM and EDX. The PtCu/CN catalyst achieves over 5.6 times the ORR mass activity and two times the stability under pulse cycling compared to commercial Pt/C. Uniquely, the study examines bimetallic NPs and local nano-sites before and after stress testing using IL-TEM. In situ analysis of PtCu/CN microstructure revealed two primary degradation mechanisms, (i) partial dissolution of NPs and (ii) NP agglomeration, with the C–N support significantly mitigating these effects through strong NP–support interactions. The findings underscore the prospects of bimetallic PtCu catalysts with nitrogen-doped support by showcasing exceptional ORR activity and durability.

## 1. Introduction

The high cost and limited availability of platinum constrains the widespread use of Pt-based materials; thus, the production of bimetallic catalysts with a reduced platinum content and superior characteristics is deemed as an urgent task in the field of hydrogen energy [1,2].

The comprehensive approach that combines this work with a composition and/or structure of bimetallic nanoparticles (NPs) and the work with a carbon support (CS) is being found in a range of studies deriving outstanding results [3,4,5,6]. The main approach to increasing the catalysts activity in the oxygen reduction reaction (ORR) is the synthesis of bimetallic NPs of various structures, including nanoframes, nanowires, core–shell, nanopores, and de-alloyed systems [3,4,7,8,9]. NPs of a special structure are characterized by a more active surface due to deformation and electronic effects [3,9,10]. Peter Strasser et al. have shown the prospects of using PtNi/C materials at the proton-exchange membrane fuel cell (PEMFC) cathode [11,12]. Numerous studies by Hodnik et al. demonstrate outstanding PtCu/C ORR characteristics [9,13,14].

To enhance the structure stability of the electrocatalysts and extend their service life, a growing body of research has recently been concerned with the use of modified carbon supports [4,15,16,17,18]. In [16], it has been shown that the introduction of heteroatoms, e.g., nitrogen, phosphorus, or sulfur, into CSs may change their electrostatic properties, which, in turn, contributes to the uniform distribution of platinum-containing NPs inhibiting the degradation processes caused by agglomeration and/or detachment of NPs from the support. Despite the efforts of researchers, the question of the bimetallic systems stability with a reduced platinum content for PEMFCs along with the simultaneous preservation of increased ORR activity is still to be addressed.

In this paper, we present a novel PtCu/C catalyst containing de-alloyed bimetallic NPs deposited on an N-doped CS as an active component. Although membrane electrode assemblies (MEAs) better replicate operational fuel cell conditions, rotating disk electrode (RDE) measurements preferred method for early-stage R&D characterization and remain essential for fundamental catalyst evaluation due to their material efficiency (>10× less catalyst needed), reduced system complexity, and superior experimental control for studies. We furthermore applied the identical location transmission electron microscopy (IL-TEM) approach to quantify and compare PtCu nanoparticles before and after accelerated stress test (AST) on RDE.

## 2. Experimental Section

**Figure 1:**
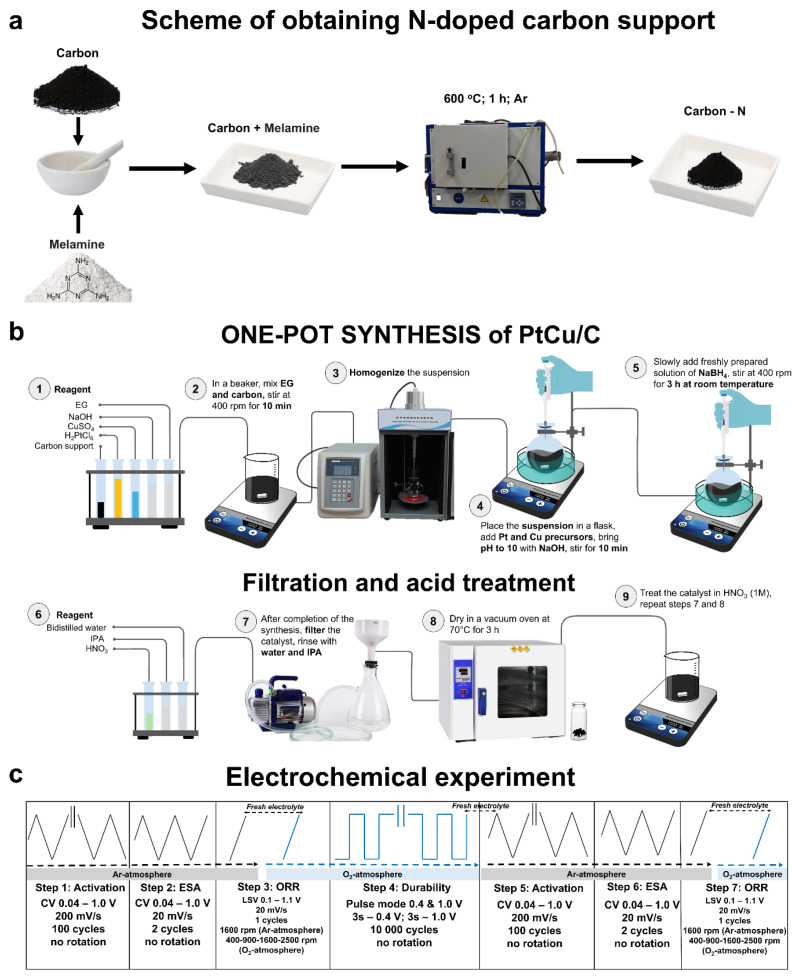
Scheme of preparing a doped CS (**a**). Scheme of synthesizing the PtCu/CN on the support (**b**). Scheme of the electrochemical experiment (**c**).

Ketjenblack-EC600JD (Nouryon Functional Chemicals LLC, Chicago, IL, USA) with a surface area of 1371 m^2^/g was chosen as the initial support for further modification. The CS modification was performed with nitrogen in a tube furnace using melamine as the precursor (Figure 1a). PtCu/CN was obtained by heterophase wet synthesis using the acid treatment (Figure 1b). Additional information on the materials and synthesis stages can be found in Supplementary Materials (SMs). The well-known commercial product—HiSPEC3000 by Johnson Mattey (Hongkong, China) (20% Pt)—was selected as the reference sample.

Information on the characterization of the composition and structure of materials is presented in the Supplementary Materials. Figure 1c shows an electrochemical study scheme that includes surface activation (Step 1, 5), cyclic voltammogram (CV) registration, which determines the electrochemically active surface area (ESA) (Step 2, 6), linear sweep voltammogram (LSV) registration to assess the ORR activity (Step 3, 7), and AST (Step 4). Additional information on the conditions of electrochemical tests and the methods to calculate functional characteristics can be found in the Supplementary Materials.

**Figure 2:**
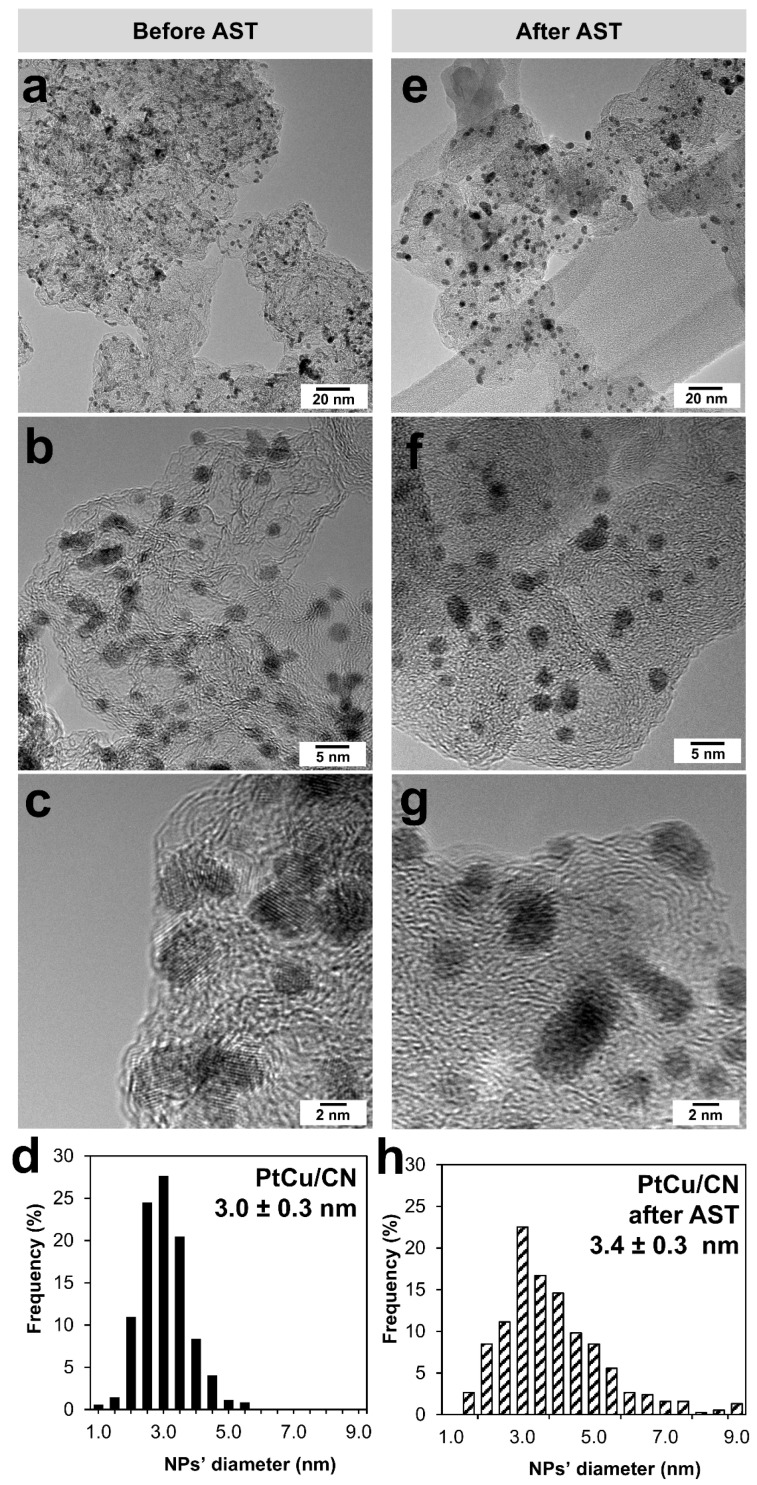
TEM images of the bimetallic catalyst before (**a**–**c**) and after (**e**–**g**) AST at the RDE. Histograms of NPs distribution in the corresponding state (**d**,**h**).

**Figure 3:**
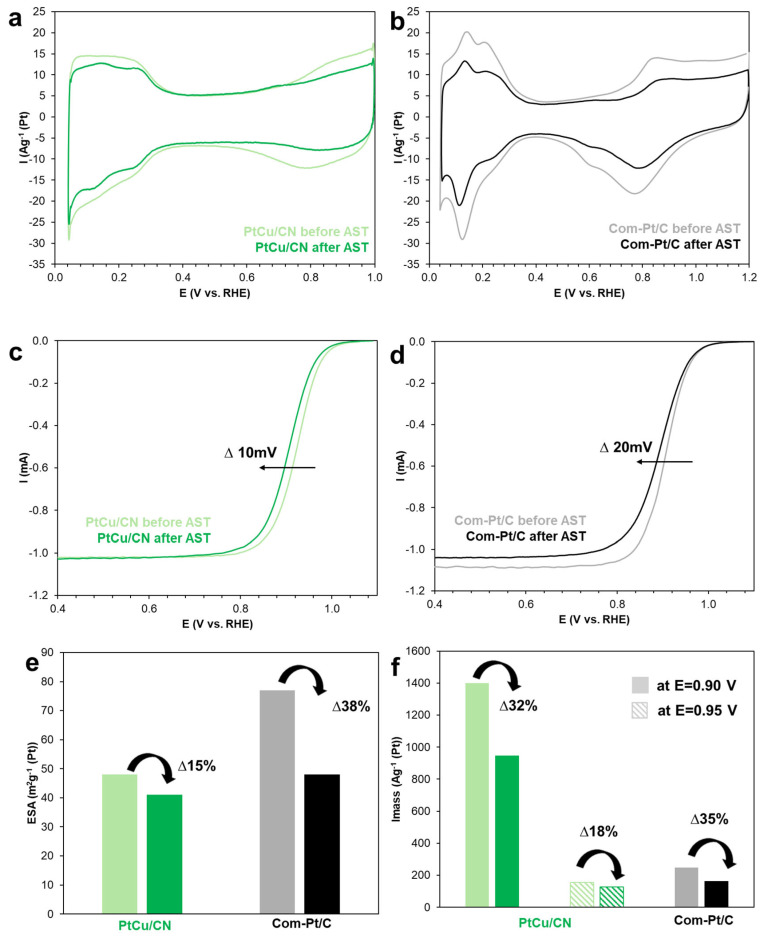
Electrocatalyst CVs in an Ar atmosphere (**a**,**b**); electrocatalyst LSVs in an O_2_ atmosphere at rotation speed of 1600 rpm (**c**,**d**). Histograms of a change in the ESA (**e**) and I_mass_ (**f**) before and after AST.

**Figure 4:**
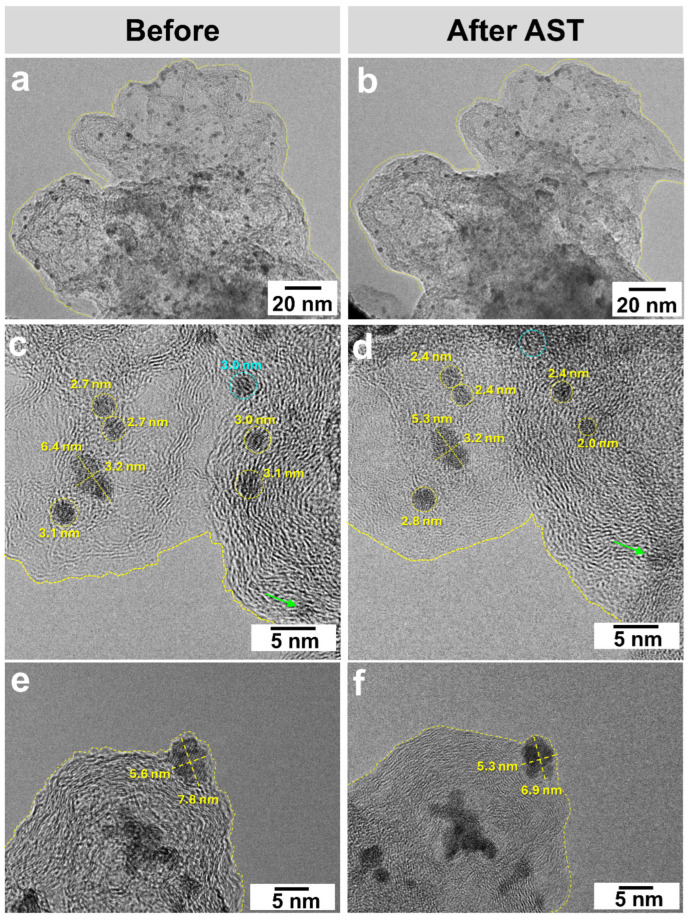
IL-TEM images of PtCu/CN before (**a**,**c**,**e**) and after (**b**,**d**,**f**) AST. Particles that dissolve are highlighted in yellow, those that migrate are highlighted in green, and those that disappear are highlighted in blue. The yellow outline highlights a change in the CS structure.

Representative IL-TEM images were acquired from catalyst regions immobilized at the corners of the uncoated gold grid (Figure S1b,c). In this case, figures 1 to 10 were selected on the grid (Figure S1a), at the corners of which TEM images of several sections of the catalyst were taken (Figure S1d). The grid was thus placed in a special Teflon holder with a platinum plate inside, fixed, placed in a three-electrode cell (Figure S1e), and subjected to the electrochemical tests (except for measuring the ESA and ORR activity values) described in Tables S1 and S2. Following the electrochemical tests, the grid was subjected to the TEM measurements at previously selected sites (Figure S1f).

## 3. Results and Discussion

The source of nitrogen atoms for doping during high-temperature treatment of a highly porous CS (KetjenBlackEC600-JD, Nouryon Functional Chemicals LLC, Chicago, IL, USA) is melamine. This method of modifying the support makes it possible to introduce nitrogen atoms into its structure up to 5 at% [19,20]. The borohydride method in the liquid phase has been chosen as the method to synthesize PtCu/C due to its simplicity, short duration, and reproducibility. Following the acid treatment, the obtained PtCu/CN catalyst with de-alloyed PtCu NPs is characterized by a platinum mass fraction of about 20% and a metal component composition of PtCu_0.3_ determined by TXRF (Table S1).

The XRD profile of the PtCu/CN sample has an appearance characteristic of platinum–carbon materials (Figure S2). The reflexes of the (111) and (200) facets are shifted to the high-angle region of 2θ relative to pure platinum (39.9° and 43.2°), which is associated with the formation of a PtCu solid solution (Figure S1) [21]. The crystallite composition calculated according to Vegard’s law is PtCu0.4, which is close to the composition of the metal component determined by total reflection X-ray fluorescence (TXRF) (Table S1). However, the composition according to Vegard’s law should be treated with caution in this regard, since for Pt/C on a doped support, we have previously observed a shift in the Pt(111) reflex towards the low-angle region of 2θ, which is due to the influence of nitrogen [22].

The PtCu/CN average crystallite size determined by the Scherrer equation for the (111) facet is less than 2 nm (Table S1). The small crystallite size may be caused by a broadening in the (111) peak due to the non-uniform distribution of metal atoms inside NPs or the insufficient NP crystallinity (Figure S1). For the monometallic Com-Pt/C, the average crystallite size is 0.6 nm larger than that of PtCu/CN (Table S1).

The TEM images of PtCu/CN exhibit small NPs (about 3 nm) uniformly distributed over the CS (Figure 2a–c). Most NPs are individual. A small proportion of NPs intersect with 1–3 “neighbors”. Since no additional stabilizing agents (except EG as a solvent) have been used in the synthesis, the agglomerates from 10 to 20 nm of indefinite form are expected to be present in the material (Figure 2a).

The support mesoropores of 5–10 nm in size are clearly visible in the high-angle annular dark-field scanning transmission electron microscopy (HAADF-STEM) image (Figure S3b). The micrograph taken by secondary electron imaging (SEI) demonstrates a small number of metal NPs localized directly on the surface of carbon microspheres (Figure S3). This means that most of the PtCu particles are localized in the micro- and mesopores of the doped CS.

The elemental analysis of separate catalyst sections performed by the energy dispersive X-ray (EDX) microanalysis confirms the bimetallic nature of NPs, with platinum and copper being localized at the same sections of the support (Figure S4a,b). The qualitative EDX in-line scanning of individual NPs is only possible for particles larger than 5 nm, since smaller particles may drift under the detector beam changing their structure. The scanning of NPs with a diameter of 9.5 nm demonstrates the presence of a platinum shell with a thickness of about 2 nm (Figure S4c).

According to the EDX results, the nitrogen content at different sections of the catalyst varies from 1.4 to 7.7 at% (Figure S5). Moreover, according to the mapping results, there is an increase in the nitrogen concentration near NPs identified in the HAADF-STEM images (Figure S5). This may stand for the uniformity of NPs distribution over the support surface. In the course of synthesis, NPs are predominantly formed and deposited on the CS active centers saturated with nitrogen atoms.

A detailed comparative analysis of the CV and LSV shapes for PtCu/CN and Com-Pt/C (Figure S6) is provided in the Supplementary Materials. The ESA calculation based on the CV data (Figure 3a,b) has shown that the area of the bimetallic catalyst is inferior to the commercial Pt/C, which correlates well with the literature data [23] (Figure 3e). The ORR mass activity at E = 0.90 V for PtCu/CN is 5.7 times higher than that of Com-Pt/C (Table S2).

It is known that nitrogen incorporated into the carbon matrix (N-doped carbon) alters the electronic and chemical properties of the support [24,25]. Nitrogen can enhance electrical conductivity by providing additional charge carriers (donor effect of pyridinic and graphitic nitrogen). Nitrogen-containing groups on the support surface create active sites (e.g., pyridinic N) capable of adsorbing metal nanoparticles. We believe that nitrogen-containing functional groups bind more strongly to PtCu, reducing their agglomeration and migration across the support surface. This stabilizes the nanoparticles and may also increase their dispersion. Additionally, nitrogen groups on the CS can modify the electronic structure of the bimetallic nanoparticles. This leads to a downshift in the Pt d-band center, weakening the binding of oxygen intermediates (*O, *OH) and accelerating the ORR. Thus, while Cu in the alloy with Pt induces compressive lattice strain, nitrogen in the support may further enhance this effect.

Maintaining the high stability of catalysts in the ORR remains an essential task in the development of novel platinum-containing materials for acidic electrolytes [26]. The durability of the catalysts studied has been assessed in a pulse mode at potentials of 0.4 and 1.0 V in an oxygen atmosphere over 10,000 cycles. The materials degradation degree has been evaluated by a change in the ESA and the ORR mass activity during AST (Figure 3). By a change in the CV shape before and after AST (Figure 3a,b), we may conclude that the currents in the hydrogen region for Com-Pt/C decrease more drastically than for PtCu/CN, which indicates a greater change in the ESA, i.e., by 38 and 15%, respectively (Figure 3e; Table S2).

For both the catalysts, we may observe a shift in E_1/2_ after AST, which is associated with the materials degradation (Figure 3c,d). The half-wave potential of PtCu/CN before and after AST is 0.94 and 0.93 V, respectively. In this regard, we consider it advisable to additionally assess the activity and its changes during AST at a higher potential value (E = 0.95 V) (Figure 3f). The degradation degree of the mass activity is 32 and 35% for PtCu/CN and Com-Pt/C at 0.90 V, respectively, and 18% for PtCu/CN at 0.95 V (Figure 3f, Table S2).

The use of AST at the RDE has allowed us to record a decrease in the electrocatalysts’ electrochemical characteristics. Obtaining data on any changes in the microstructure of samples after AST is an important step for understanding the degradation processes and the possibility of their prevention.

After the stress testing, we conducted a TEM analysis for changes in the PtCu/CN microstructure in two ways: (1) the catalyst layer was removed from the RDE after AST (ex situ) (Figure 2e–g and Figure S7); (2) after the catalyst was deposited onto a golden patterned grid, the characteristics were studied before and after AST at the same local sections of the sample, i.e., by IL-TEM (quasi in situ) (Figure 4 and Figure S8).

In the event of processing PtCu/CN various sections in the ex situ TEM images before and after stress testing, we may observe an enlargement in the particle size of 0.4 nm as a result of the AST (Figure 2h). The NP spatial distribution remains fairly uniform, with a large proportion of NPs having a size of 3.0 nm (Figure 2e–h). At the same time, there is a change observed in the nature of the NP size distribution; i.e., the proportion of particles with a size of 1.0–3.5 nm decreases, the proportion of particles with a size of 4.0–5.5 nm grows, and the width of the size dispersion increases up to 9 nm (Figure 2h). In this regard, the dissolution of small-sized particles and the redeposition of metal atoms into larger NPs (Ostwald ripening) as well as the agglomeration of NPs resulting from their migration over the support surface appear to be the prevailing mechanisms. A slight increase in the average size of PtCu/CN NPs leads to an insignificant loss in the ESA during AST (Figure 3e, Table S2).

The EDX mapping performed after AST confirms the preservation of the alloying component in PtCu/CN NPs. The scanning of NPs of 8.9 nm confirms the presence of a Pt-shell with a thickness of about 2 nm (Figure S7c), which is comparable to the scanning results before AST (Figure S4c).

The transition to IL-TEM for studying changes in the catalyst during AST is necessary for a deeper understanding of the catalyst degradation mechanisms. When using IL-TEM, we may clearly observe a change in the carbon morphology in the corresponding images, which may be caused by its oxidation (Figure 4 and Figure S8). Most spherical NPs with a diameter of 2.2 to 3.9 nm decrease in size by up to 30% (Figure 4c–f and Figure S8c,d). At the same time, the decrease in size is typical for both spherical and rod-shaped NPs (Figure 4c,d). The PtCu/CN TEM images before AST have revealed the particles that are absent in the same after AST (Figure 4c,d and Figure S8c,d). The disappearance of NPs is associated with their dissolution or detachment from the support surface. It is not possible to separate the contributions of these processes by 2D images. Along with the decrease in diameter, we may observe the process of NP agglomeration (Figure 4c,d and Figure S8c,d). We have also managed to record the migration of NPs with an increase in the particle size (Figure S8c,d).

The analysis of IL-TEM results presents significant challenges in interpretation. The key issues include the inability to conduct EDX measurements at the same sections due to interference from the golden grid, which complicates qualitative signal collection from the catalyst. A prospective solution involves the use of a carbon-coated grid, although this coating may degrade during electrochemical testing. Additionally, plotting histograms of NP size distribution (Figure S8e,f) necessitates processing numerous micrographs with precise approximation—a task made difficult by the expected detachment of the catalyst sections after prolonged electrochemical measurements, making IL-TEM a time-consuming approach for data collection.

Despite these challenges, several advantages of the IL-TEM method are noteworthy. It is known that the dissolution of bimetallic NPs is greater for those smaller than 1.5 nm [27]. The IL-TEM analysis has revealed a reduction in the size of larger NPs, specifically those with diameters between 2 and 4 nm. Furthermore, post-AST images indicate that the support exhibits only minor structural changes, highlighting its enhanced resistance to degradation—a finding that would not be apparent without IL-TEM.

In summary, the IL-TEM analysis revealed two primary degradation pathways:

(i) Partial nanoparticle dissolution: Sub-2 nm NPs dissolved preferentially due to their high surface energy. Larger NPs (2–4 nm) shrank by up to 30%, indicating surface atom dissolution (Figure 4 and Figure S8).
(ii) Aggregation and Ostwald ripening: NP migration and coalescence occurred but were less pronounced than dissolution. We attribute the suppressed aggregation to the N-carbon support, mediated by strong N-PtCu interactions (EDX mapping, Figure S5).

Apparently, the nitrogen-doped support affects degradation through an anchoring effect—pyridinic/graphitic nitrogen serve as nucleation centers, immobilizing NPs and reducing their migration. This explains the lower ESA loss (15%) for PtCu/CN compared to Pt/C (38%) after AST. There is also an electronic effect whereby nitrogen weakens the Pt-O bond, reducing dissolution. Note that dissolved Pt^2+^/Cu^2+^ ions may randomly redeposit onto the support as atoms and clusters, remaining undetected by IL-TEM.

The combined use of TEM and IL-TEM offers comprehensive insights into microstructural changes after AST with consistent results. Notably, there is no reliable evidence supporting Ostwald ripening at the studied PtCu/CN local sections; instead, the observed increase in NPs sizes appears to stem from agglomeration. This process is minimal, as indicated by the stable ESA and higher ORR mass activity after AST. However, one cannot dismiss the possibility of atomic platinum dissolution and redeposition onto the CS, which is undetectable via TEM or IL-TEM.

## 4. Conclusions

The platinum–copper NPs were synthesized on nitrogen-doped carbon supports via the borohydride method. The approach can be deemed technologically facile to implement in larger-scale production.

The catalyst exhibits a high degree of NP distribution uniformity, which is challenging with unmodified carbon and surfactants. Nitrogen atoms create localized clusters acting as active centers for NP deposition, as confirmed by HAADF-STEM and EDX.

The PtCu/CN catalyst achieves a 5.6-times-greater ORR mass activity compared to the commercial Pt/C catalyst. The PtCu/CN stability under pulse cycling is twice as high as that of Com-Pt/C. Thus, the uniform distribution of metal NPs significantly increases ESA, ORR activity, and stability under stress testing conditions.

Using highly efficient IL-TEM, the study identifies partial NP dissolution and agglomeration as the key degradation mechanisms of PtCu/CN.

The results of this study highlight the success in the development of a bimetallic catalyst based on PtCu NPs using an N-doped support, which demonstrates impressive activity and durability in the ORR. The prospects for further research include optimizing the catalyst composition, investigating the mechanisms of interaction between nitrogen and metal NPs, and optimization in MEAs, which would confirm its effectiveness for scalable implementation in fuel cell technologies and other electrochemical systems.

## Data Availability

Data are contained within the article and Supplementary Materials.

## Supplementary Materials

The following supporting information can be downloaded at: https://www.mdpi.com/article/10.3390/nano15100776/s1, Figure S1. Schematic representation of the IL-TEM method application: the catalytic ink is applied to the patterned grid (a); a catalyst site is selected in the corner of the grid in numbers (b,c); the TEM measurements are performed before accelerated stress testing (AST) (d); the grid is placed in a three-electrode cell and subjected to AST (e); the TEM measurements are performed at the same section of the catalyst after AST (f). Table S1. Composition and structural parameters of the PtCu/C and commercial Pt/C catalysts. Table S2. Electrochemical characteristics of the catalysts before and after AST. Figure S2. XRD patterns of the bimetallic catalyst. XRD patterns of carbon Ketjenblack-600JD before and after heat treatment with melamine (insert). Figure S3. BF-STEM (a), HAADF-STEM (b), and SEI (c) images of the PtCu/CN sample in the “as-prepared” state. Figure S4. HAADF-STEM images of the bimetallic catalyst before AST and EDX maps of the selected section: Cu, Pt (a). HAADF-STEM with EDX maps (b) and in-line scanning (c) of individual NPs. The green arrow (b) indicates a direction of scanning. Figure S5. HAADF-STEM images of the bimetallic catalyst before AST (a,c,e) and EDX maps of nitrogen (b,d,f) at the corresponding sections. Nanoparticles are outlined in yellow and their stencil is transferred to the nitrogen maps. Figure S6. Electrocatalysts CVs in an Ar atmosphere at the stationary electrode (a); electrocatalysts LSVs in an O_2_ atmosphere at an RDE rotation speed of 1600 rpm (b). 0.1 M HClO_4_ as the electrolyte, potential scanning rate 20 mV/s. I^−1^–ω^−0.5^ dependence graphs at a potential of 0.90 V (insert in Figure b). Figure S7. HAADF-STEM images of the bimetallic catalyst after AST at the RDE and EDX maps of the selected section: Cu, Pt (a). HAADF-STEM with EDX maps (b) and in-line scanning (c) of individual NPs. The green arrow (b) indicates the direction of scanning. Figure S8. IL-TEM images of the bimetallic catalysts before (a,c) and after (b,d) AST. Histograms of NPs distribution in the corresponding state (e,f). Particles that dissolve are highlighted in yellow, those that migrate are highlighted in green, and those that disappear are highlighted in blue. The yellow outline highlights a change in the CS structure. Refs. [22,28,29,30,31,32,33] are cited in the supplementary materials.
