# Supplementary material for: Enhanced Electrocatalysts for Oxygen Reduction Reaction: Insights from Accelerated Stress Testing and IL-TEM Analysis
Source: Nanomaterials (Basel). 2025 May 21;15(10):776. doi: 10.3390/nano15100776 (PMC12114201; doi:10.3390/nano15100776)
Supplement: Supplementary file 1 [file nanomaterials-15-00776-s001.zip › nanomaterials-3656591-supplementary.pdf]

Supplementary Materials

Enhanced Electrocatalysts for Oxygen Reduction Reaction: Insights from Accelerated Stress Testing and IL-TEM Analysis

Angelina S. Pavlets [1], Elizaveta A. Moguchikh [1], Ilya V. Pankov [2], Yana V. Astravukh [1], Sergey V. Belenov [1] and Anastasia A. Alekseenko [1,*]

[1] Faculty of Chemistry, Southern Federal University, 7 Zorge St., Rostov-on-Don 344090, Russia
[2] Research Institute of Physical Organic Chemistry, Southern Federal University, 194/2 Stachki St., Rostov-on-Don 344090, Russia

1. Materials and reagents

The following chemicals and materials were used in the experimental work: carbon support KetjenblackEC600-JD (Cabot Corporation), ethylene glycol (top grade, not less than 99.8%, Rehacor, LLC), $H_2PtCl_6{\cdot}6H_2O$ (TU 2612-034-00205067-2003, mass fraction of Pt 37.6%, Aurat, Russia), $CuSO_4{\cdot}5H_2O$ (JSC Vekton), sodium hydroxide (Rehacor, LLC), $NaBH_4$ (JSC Vekton), HNO_3 (JSC Vekton), bidistilled water (conductivity <5 μS/cm), $HClO_4$ (chemically pure, 66%, Vekton, Russia), isopropanol (extra-pure grade, Vekton, Russia), 10% solution of Nafion® D1021 (Dupont, Sigma Aldrich, USA), and gaseous argon (99.998%, Research Institute of Construction Materials, Russia).

2. Preparation of nitrogen-doped carbon support

A mixture of carbon and melamine in a ratio of 1:5 was placed in a furnace (PKT 14, Teplopribor LLC), and the atmosphere was saturated with argon at room temperature for 30 min. After that, the support was synthesized at a temperature of 600 °C (heating rate 20°C/min) and a gas flow rate of 0.2 cm^3/s for 60 min. The C–N material was synthesized in an inert atmosphere (Ar). According to our previous work, when using this doping method, the nitrogen content in the support is about 5 at.% [1].

3. Method to synthesize the catalyst

0.3 g of the support were dispersed by ultrasound in 120 mL of ethylene glycol (Figure 2, Steps 2–3). Aqueous solutions of hexachloroplatinic acid and copper sulfate precursors were added to the suspension in order to obtain a catalyst with a 20% platinum mass fraction and a composition of Pt:Cu = 1:1 (Figure 2, Step 4). The system pH was adjusted to 10 using a 1 M NaOH solution (Figure 2, Step 4). Then, a freshly prepared 0.5 M $NaBH_4$ solution (reducing agent), which was taken in a threefold excess relative to the metals, was slowly added to the suspension, and the reaction mixture was allowed to stand with stirring for 3 h at room temperature (Figure 2, Step 5). The obtained catalyst was separated by filtration, rinsed with isopropanol and bidistilled water (Figure 2, Step 7), and dried in a vacuum drying cabinet at a temperature of 70 °C for 3 h (Figure 2, Step 8). The resulting catalyst was acid-treated in 1 M HNO_3 for 3 h (Figure 2, Step 9), followed by filtration and drying as described above.

The PtCu/CN electrocatalyst was synthesized by a heterophase wet synthesis method by employing an acid treatment process. Initially, 0.3 g of the support material were ultrasonically dispersed in 120 mL of ethylene glycol (Figure 2, Steps 2–3). Next, aqueous solutions of hexachloroplatinic acid and copper sulfate precursors were added to the suspension, aiming to achieve a platinum mass content of 20% with a Pt:Cu ratio of 1:1 (Figure 2, Step 4). The system pH was thus adjusted to 10 using a 1 M NaOH solution (Figure 2, Step 4).

Subsequently, a freshly prepared 0.5 M $NaBH_4$ solution, acting as the reducing agent, was slowly introduced in a threefold excess relative to the metal content. The reaction mixture was allowed to stand with stirring for 3 h at room temperature (Figure 2, Step 5). The resulting catalyst was separated by filtration, rinsed with isopropanol and bidistilled water (Figure 2, Step 7), and dried in a vacuum drying cabinet at 70 °C for 3 h (Figure 2, Step 8). Finally, the obtained catalyst was subjected to acid treatment in 1 M HNO_3 for 3 h (Figure 2, Step 9), after which it was filtered and dried as previously described.

Scheme 1c represents specific stages of the synthesis conducted, which include the suspension composition (Step 1), its preparation and further dispersion (Steps 2, 3), pH adjustment and addition of the reducing agent (Step 4, 5), filtration (Step 6, 7), drying (Step 8), and subsequent acid treatment of the resulting material (Step 9).

4. Study of the catalyst composition and structure

4.1 Brunauer–Emmett–Teller (BET) measurements

Measurements of nitrogen adsorption isotherms at 77 K are carried out using an automated computer-aided device (Autosorb-6B by Quantachrome Instruments (USA)). The device consists of two independent units. The first unit is designed for sample preparation, in which they are heated in dynamic vacuum using a forevacuum pump and a furnace, where the temperature is adjusted from 20 to 350 °C with an accuracy of 1 °C. The preparation is considered completed when reaching a stable residual pressure of less than 5×10^{-2} Torr measured by a thermocouple vacuum gauge.

Following the preparation, the ampoules are cooled to room temperature, filled with ultra-high purity nitrogen to atmospheric pressure, and transferred to a second unit for measuring adsorption isotherms. This unit simultaneously measures isotherms for two samples using two ports and capacitance pressure sensors. Measurements are carried out by a volumetric method with a periodic dosage rate for nitrogen gas. To minimize errors associated with a change in ambient conditions, the saturated vapor pressure at liquid nitrogen temperature is continuously measured using a nitrogen thermometer, which takes measurements at standard time intervals.

4.2 Gravimetry analysis

The metals mass fraction in the electrocatalysts was determined by gravimetry from the mass of the unburned residue when heated to 800 °C, the oxidation of copper to CuO being taken into account. The UED-7-10D muffle furnace (UED Group, Saint Petersburg, Russia) with the possibility of heating up to 1,000 °C was used for the analysis.

4.3 X-ray fluorescence (XRF) analysis

To determine the metals ratio in the samples, the method of X-ray fluorescence analysis on the RFS-001 spectrometer with the total external reflection of X-ray radiation (Research Institute of Physics, Southern Federal University, Rostov-on-Don) was used. The samples exposure time was 300 s. The registration and processing of the X-ray fluorescence spectra were performed using the UniveRS software (Southern Federal University, Rostov-on-Don). The resulting accuracy for the obtained X-ray fluorescence spectra was ±0.1.

4.4 X-ray powder diffraction (XRD) analysis

The X-ray diffraction patterns were recorded at room temperature using the ARL X`TRA powder diffractometer (Thermo Scientific, Switzerland) with the Bragg-Brentano geometry (θ-θ) and the filtered CuKα radiation (λ = 0.154056 nm). The materials X-ray diffraction patterns were recorded in the 2θ angle range of 15–55°. The fitting of the X-ray diffraction patterns with the SciDAVis software was performed using the Lorentz function. The results of the approximation and the separation of contributions from different reflections were used in further calculations.

The average crystallite size of the metal phase (D_{av}) was determined by the Scherrer equation for a more intense peak (111). Notably, the particle size determined by the Scherrer formula was compliant with the average crystallite size (coherent scattering regions). This parameter calculated from the full width at half maximum (FWHM) of the peak (111) for the PtCu/C materials should be treated with caution, since this peak might actually be a superposition of the reflections of two phases based on copper and platinum. The accuracy of determining D_{av} was ±5%.

4.5 TEM, EDX, and SEI analyses

Transmission electron microscopy (TEM), bright field (BF) and high-angle annular dark-field (HAADF) scanning transmission electron microscopy (STEM) images were obtained using the JEOL JEM-F200 microscope operating at an accelerating voltage of 200 kV.

The JEOL EM-01361RSTHB dual tilt beryllium holder was used for the TEM, secondary electron imaging microscopy (SEI) and energy-dispersive X-ray spectrometry (EDX) measurements. The EDX analysis was performed with the Bruker Xflash 6T/60 Quantax 400-STEM system.

To perform the measurements, 0.5 mg of the catalyst were placed in 1 mL of isopropanol and dispersed with ultrasound. A drop of the resulting suspension was applied to the copper grid covered with a layer of amorphous carbon, which was dried in air at room temperature for 20 min. The TEM images were recorded at magnifications ranging from 30,000x to 800,000x using 200 μm and 100 μm condenser apertures over an exposure range of 500 to 1,000 ms using the high-resolution CMOS AMT camera.

The histograms of the nanoparticles size distribution in the catalysts were plotted, taking into account the sizes of at least 300 particles detected and randomly selected in the TEM images.

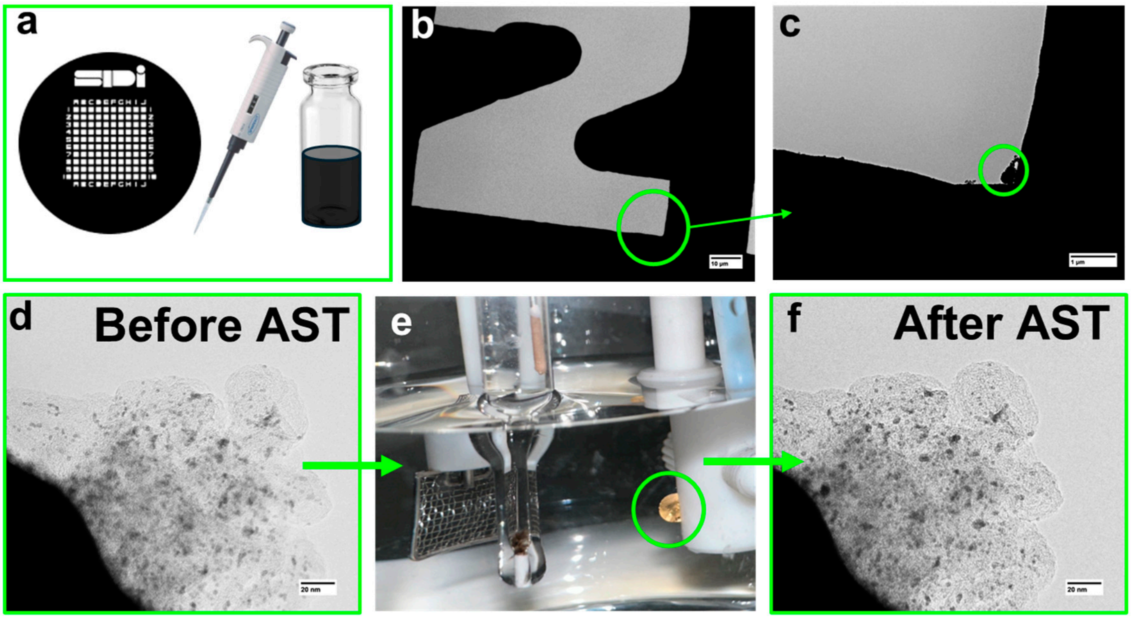

Figure S1. Schematic representation of the IL-TEM method application: the catalytic ink is applied to the patterned grid (a); a catalyst site is selected in the corner of the grid in numbers (b–c); the TEM measurements are performed before accelerated stress testing (AST) (d); the grid is placed in a three-electrode cell and subjected to AST (e); the TEM measurements are performed at the same section of the catalyst after AST (f).

5. Study of the catalysts electrochemical characteristics

5.1 Method to prepare the catalytic ink

The catalyst suspension (catalytic ink) was obtained by adding 1,500 μL of isopropyl alcohol, 500 μL of deionized water, and 40 μL of a 5% aqueous alcoholic Nafion® D520 emulsion to a 0.0040 g of the catalyst sample studied. The suspension was then dispersed in an ultrasonic bath for 10 min, after which it was stirred for 5 min on a magnetic stirrer. After stirring, it was repeatedly dispersed in an ultrasonic bath for 10 min and stirred for 5 min on a magnetic stirrer. The water temperature in the ultrasonic bath was not to exceed 20 °C. The total preparation time for the homogeneous suspension was 30 min. Before applying the suspension, the glass–carbon end face of the rotating disk electrode (RDE) was polished using a polishing paste (Allied aluminum oxide suspension), after which, to remove the paste residues from the electrode surface, it was treated with ultrasound and rinsed with deionized water. The prepared catalytic ink was used to apply a thin layer of the catalyst onto the glass–carbon electrode.

5.2 Method to form a thin catalytic layer at the end face of the RDE

An aliquot of the catalytic ink with a volume of 2 μL was sampled using a pipette tip with continuous ink stirring. A drop of the catalytic ink of the above volume was applied to the end face of the polished and

degreased glass–carbon electrode with a diameter of 5 mm (area 0.196 cm^2). After applying the ink drop, the electrode was subjected to rotation at 700 rpm until the drop dried completely (within 10–15 min). After the first drop of the catalytic ink had dried, the second one of the required volume was applied, which was calculated in order that the total platinum loading at the RDE end face ranged from 19.0 to 21.0 μg/cm^2. Therefore, the electrode studied represented a homogeneous catalytic layer applied to the RDE end face.

5.3 Measurement of the electrochemically active surface area by adsorption/desorption of a hydrogen monolayer

Before measuring the materials electrochemical characteristics in a three-electrode electrochemical cell, it was necessary to pre-activate their surface. 0.1 M $HClO_4$ saturated with an inert gas (Ar) for 30 min was used as the electrolyte. To perform the activation, we recorded cyclic voltammograms (CVs) in the potential range of 0.04–1.00 V with a potential sweep rate of 200 mV/s during 100 cycles. Following the activation, the electrolyte was replaced with a fresh one and bubbled with inert gases for 40 min. The CVs were recorded in the potential range of 0.04–1.00 V with a potential sweep rate of 20 mV/s during 2 cycles. The electrochemically active surface area (ESA) was calculated from the obtained voltammograms by a charge amount consumed for the desorption Q_{des} and adsorption Q_{ads} of hydrogen according to the formula (S1) [2]:

$$ESA = \frac{0.5 * (Qads+Qdes)}{m(Pt)*210} \quad (S1),$$

where m_{Pt} is the metal loading at the electrode and

210 μC/cm^2 is the charge amount consumed for the hydrogen adsorption on 1 cm^2 of the Pt surface.

5.4 Measurement of activity in the oxygen reduction reaction

To determine the catalysts activity in the oxygen electroreduction reaction (ORR), a series of linear sweep voltammograms (LSVs) in the potential range from 0.1 to 1.1 V were measured at a sweep rate of 20 mV/s. The electrolyte was pre-oxygenated for 40 min while rotating the electrode at a speed of 700 rpm. To account for the ohmic voltage drop contribution, the potential of the electrode studied was calculated using the formula (S2):

$$E(RHE) = E_{set} + E_{ref} + E_{pH} - iR \quad (S2),$$

where E_{set} is the potential set value, V;

E_{ref} is the reference electrode potential, V;

E_{pH} is the adjustment for pH of the solution; and

iR is the ohmic potential drop equal to the product of the current strength by the resistance of the cell.

The resistance (R) of the assembled cell was determined by the impedance method at a voltage of 0 V relative to the reference electrode, in the frequency range of 100 kHz – 1 Hz with an amplitude of 20 mV.

The contribution of the processes occurring at the electrode in a deoxygenated solution (Ar atmosphere) was taken into account by subtracting, at a set potential, from the currents of the voltammogram obtained in an oxygen atmosphere the currents of a similar curve recorded at the same electrode during measurements in an Ar atmosphere at a rotation speed of 1,600 rpm (S3):

$$I = I(O_2) - I(Ar) \quad (S3),$$

The voltammograms were recorded at RDE rotation speeds of 400, 900, 1,600, and 2,500 rpm. The kinetic current (i) and the half-wave potential ($E_{1/2}$) were used as the indicators of ORR activity. The calculation of the current density (j_k) at a potential of 0.90 V was carried out according to the Koutetsky−Levich equation [3]. By normalizing the current density to the RDE surface area (S_{geom}), the kinetic current (i_k) was determined. Using the obtained values of i_k, the mass activity (I_{mass}) and the specific activity (I_{sp}) were further determined by recalculating the kinetic current value by the mass of platinum contained at the electrode and recalculating the mass–current value by the ESA.

5.5 Determination of the electrocatalysts stability by the method of multiple rectangular pulses in an argon atmosphere

The catalysts stability was assessed by accelerated stress testing (AST) based on repeated superposition of rectangular pulses at potentials of 0.4 and 1.0 V with an exposure time of 3 s at each value, as described in

[4]. A total of 10,000 cycles were recorded. The measurements were carried out in 0.1 M $HClO_4$ saturated with argon at room temperature. To study the degree of degradation for the materials studied, we recorded cyclic and linear sweep voltammograms before and after the stress testing. The degree of degradation (**DD**) was assessed by a change in the ESA, kinetic current, and mass activity at the end of the stress testing using Formulas S4 and S5, respectively:

$$DDESA = \frac{ESA^0 - ESA^{10,000}}{ESA^0} * 100\% \quad \text{(S4)},$$

where $ESA^{10,000}$ is the electrochemically active surface area of platinum after the accelerated stress testing and

ESA^0 is the platinum initial electrochemically active surface area.

$$DDi_{mass} = \frac{{i_{mass}}^0 - {i_{mass}}^{10,000}}{{i_{mass}}^0} * 100\% \quad \text{(S5)},$$

where ${i_{mass}}^{10,000}$ is the mass activity after the accelerated stress testing and

${i_{mass}}^0$ is the initial mass activity (before the stress testing).

6. Results and discussion

Table S1. Composition and structural parameters of the PtCu/C and commercial Pt/C catalysts.

Sample	ω(Pt), %	Composition (TXRF)	D_{cr}, nm (XRD)	D_{av}, nm (TEM)
**PtCu/CN**	20.8	$PtCu_{0.3}$	1.9	3.0
**Com-Pt/C**	20.0	-	2.5	3.0

Table S2. Electrochemical characteristics of the catalysts before and after AST.

Material	Initial				After ST					
	ESA, m^2/g (Pt)	$E_{1/2}$, V	I_k, mA	I_{mass}, A/g (Pt)	ESA, m^2/g (Pt)	$E_{1/2}$, V	I_k, mA	I_{mass}, A/g (Pt)	DD (ESA), %	DD (I_{mass}), %
**PtCu/CN**	48	**0.94**	**0.8***	**156***	41	0.93	**0.7***	**128***	15	18
			**7.1**	**1,398**			**4.8**	**947**		32
**Com-Pt/C**	77	0.91	**1.3**	**249**	48	0.89	**0.89**	**163**	38	35

*Values at E=0.95 V

According to the results of the X-ray phase analysis for the carbon supports, we may observe a shift of the C(002) facet reflex to the high-angle region of 2θ for KB600–N compared to the initial KB600 support, which is associated with an increase in the degree of graphitization when nitrogen is embedded into the graphite lattice (Figure S1 – insert) [5]. The said effect can also be observed due to high-temperature treatment of the support.

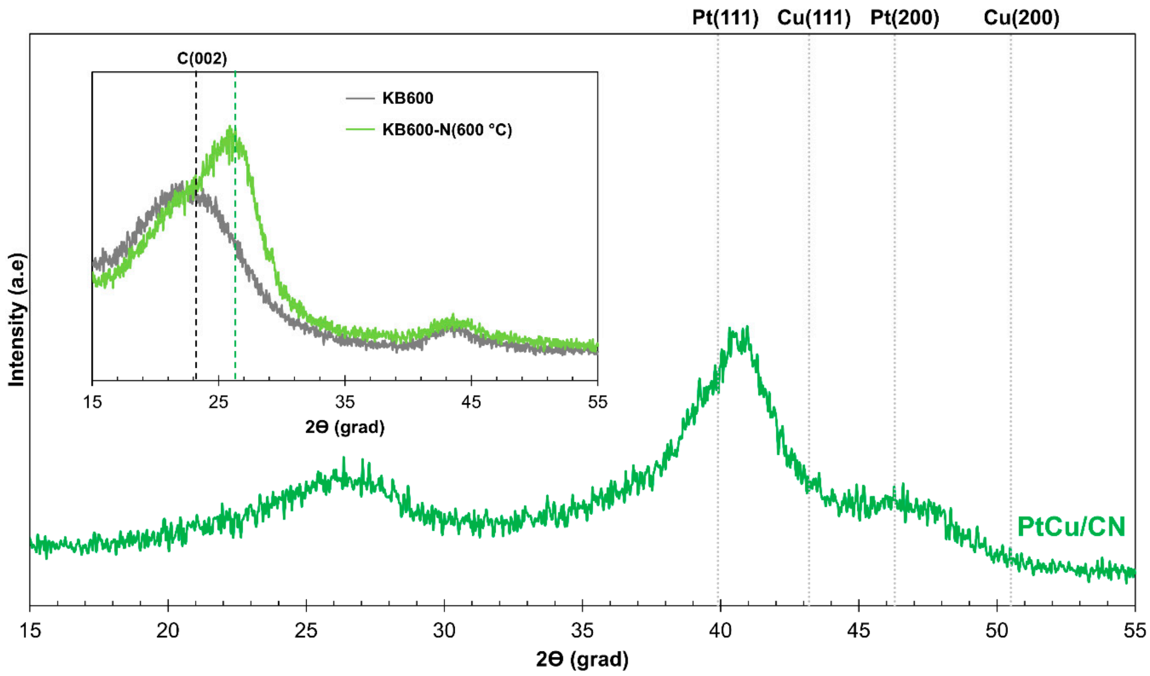

Figure S2. XRD patterns of the bimetallic catalyst. XRD patterns of carbon Ketjenblack-600JD before and after heat treatment with melamine (insert).

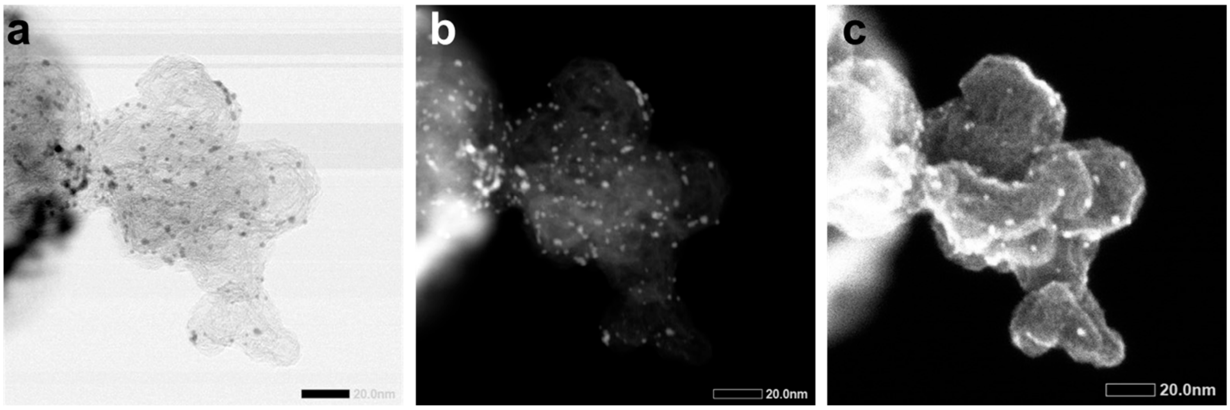

Figure S3. BF-STEM (a), HAADF-STEM (b), and SEI (c) images of the PtCu/CN sample in the "as-prepared" state.

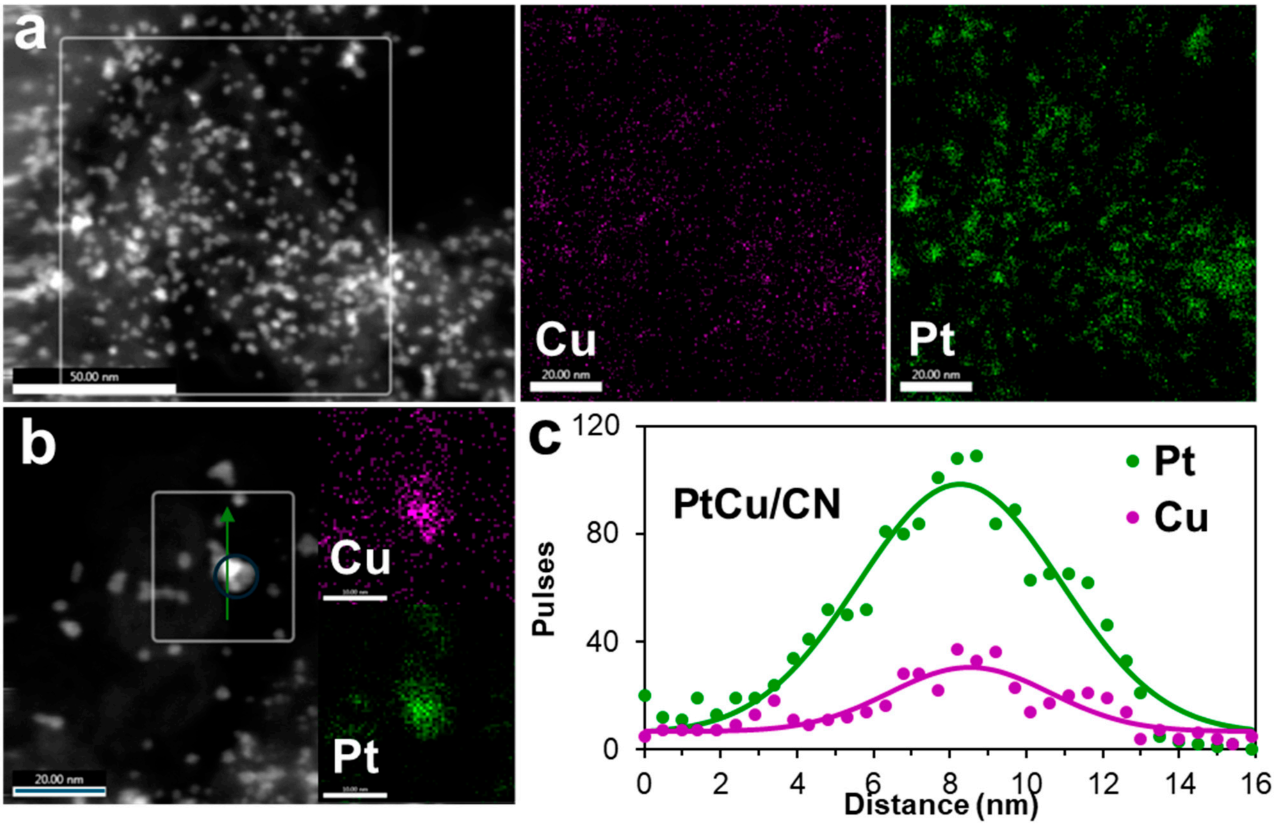

Figure S4. HAADF-STEM images of the bimetallic catalyst before AST and EDX maps of the selected section: Cu, Pt (a). HAADF-STEM with EDX maps (b) and in-line scanning (c) of individual NPs. The green arrow (b) indicates a direction of scanning.

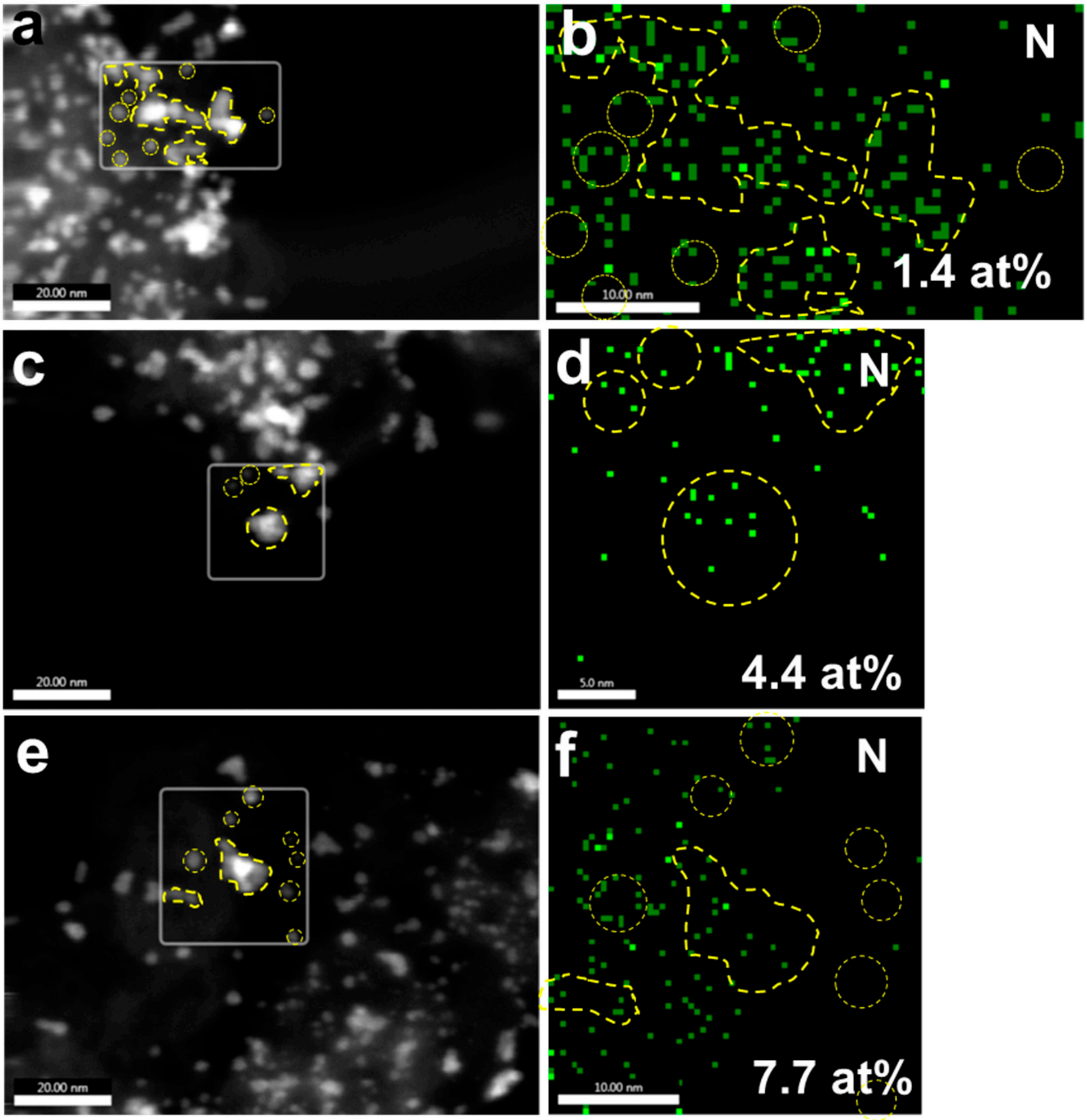

Figure S5. HAADF-STEM images of the bimetallic catalyst before AST (a, c, e) and EDX maps of nitrogen (b, d, f) at the corresponding sections. Nanoparticles are outlined in yellow and their stencil is transferred to the nitrogen maps.

For both the PtCu catalyst and the commercial platinum–carbon analog, the CVs have an appearance typical for a platinum electrode in an acidic electrolyte. For the PtCu/CN material, the currents in the double-layer region of the CVs are higher than those for Com-Pt/C (Figure S5a). Since the currents in this region are associated with non-faradaic processes of the double electric layer charge/discharge, their difference may be due to the fact that the materials are obtained on different carbon supports. The area of the support used to produce the PtCu/CN catalyst and determined by the BET method is 1,295 m^2/g. According to literature data, the Vulcan-XC72 support is used for synthesizing Com-Pt/C [6], which is characterized by a BET area of about 220 m^2/g [7]. The currents in the hydrogen region of the PtCu/CN CVs are lower than those for Com-Pt/C (Figure S5a).

The analysis of slopes in the dependences in the Koutetsky–Levich coordinates indicates that for the catalysts studied, the reaction proceeds according to the four-electron mechanism characteristic of platinum (Figure S5b – insert). The LSV of the Com-Pt/C commercial catalyst is shifted by 30 mV towards lower potential values compared to PtCu/CN (Figure S5b).

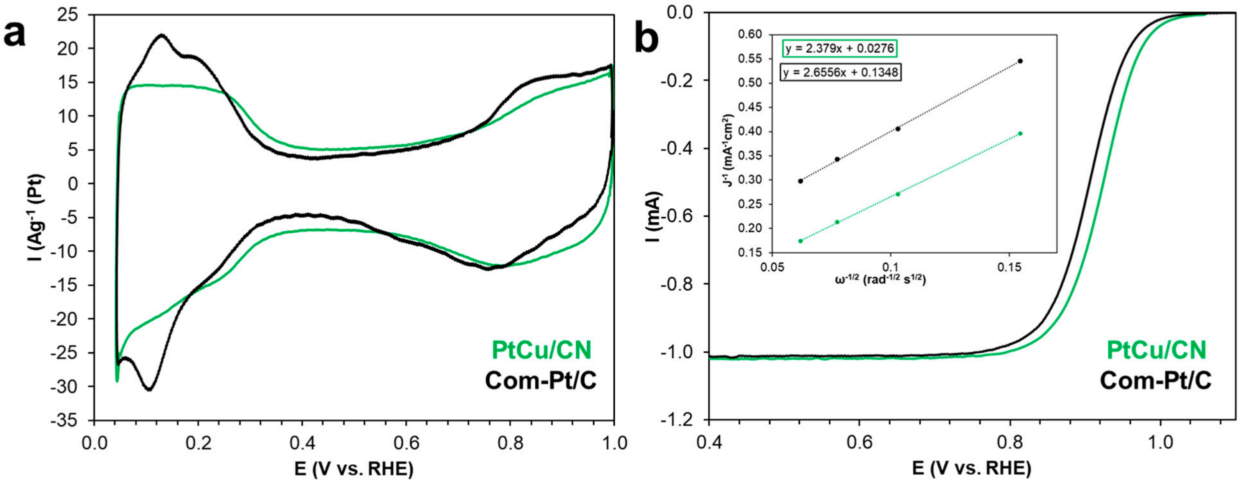

Figure S6. Electrocatalysts CVs in an Ar atmosphere at the stationary electrode (a); electrocatalysts LSVs in an O_2 atmosphere at an RDE rotation speed of 1,600 rpm (b). 0.1 M $HClO_4$ as the electrolyte, potential scanning rate 20 mV/s. $I^{-1} - \omega^{-0.5}$ dependence graphs at a potential of 0.90 V (insert in Figure b).

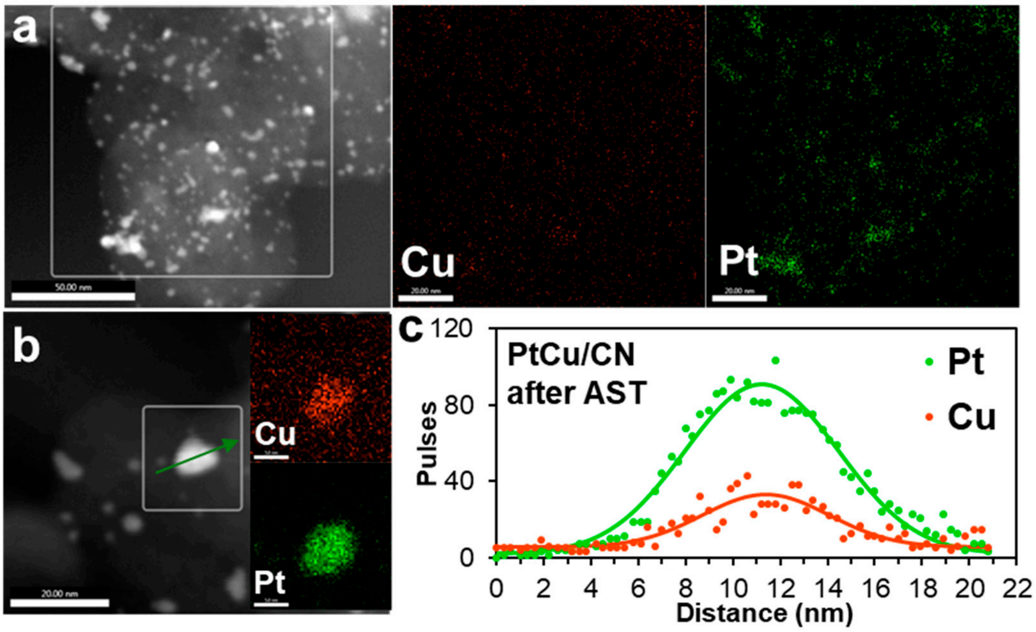

Figure S7. HAADF-STEM images of the bimetallic catalyst after AST at the RDE and EDX maps of the selected section: Cu, Pt (a). HAADF-STEM with EDX maps (b) and in-line scanning (c) of individual NPs. The green arrow (b) indicates the direction of scanning.

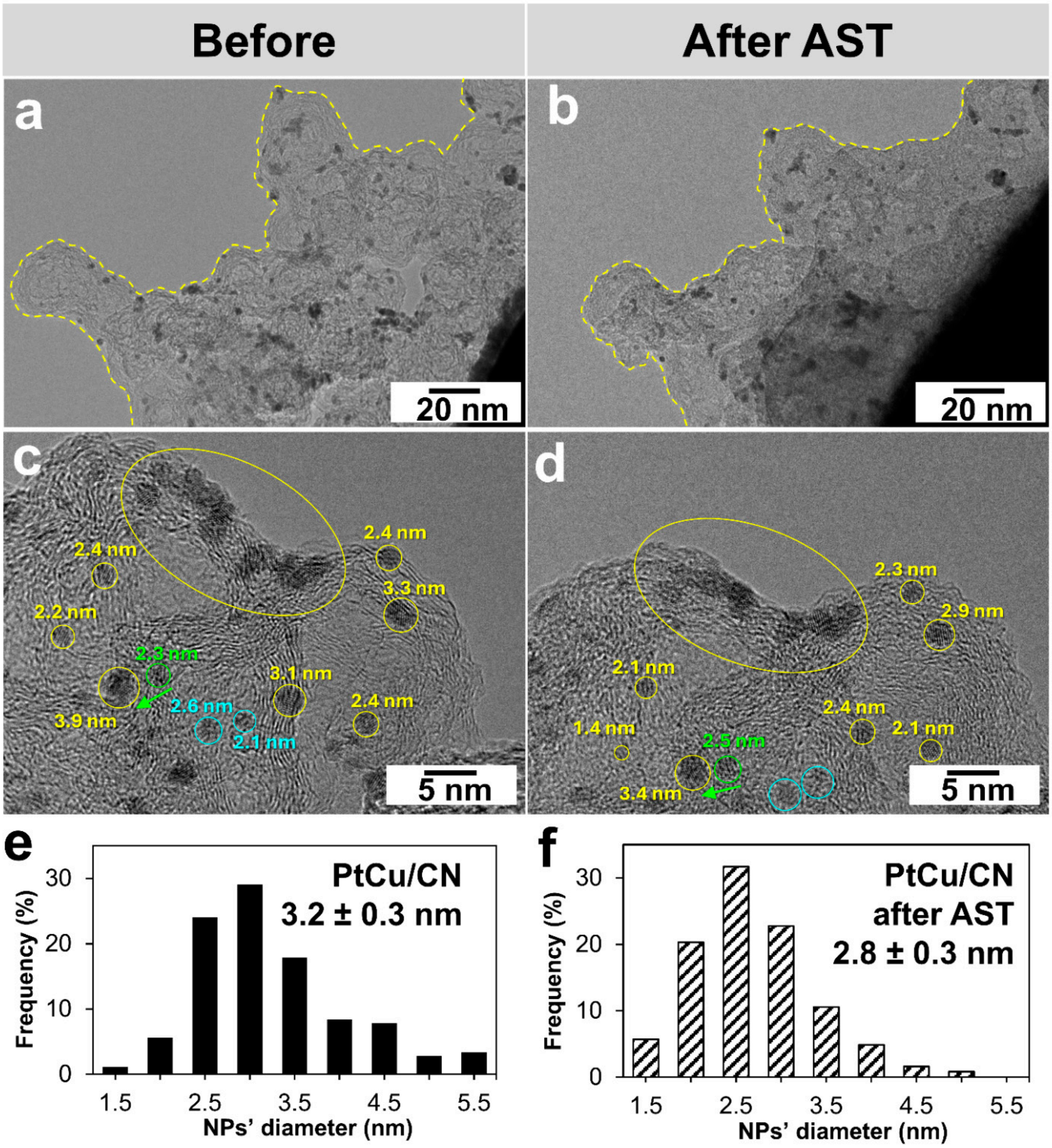

Figure S8. IL-TEM images of the bimetallic catalysts before (a, c) and after (b, d) AST. Histograms of NPs distribution in the corresponding state (e, f). Particles that dissolve are highlighted in yellow, those that migrate are highlighted in green, and those that disappear are highlighted in blue. The yellow outline highlights a change in the CS structure.

References

1. Nevelskaya, A.K.; Belenov, S. V.; Pavlets, A.S.; Menshikov, V.S.; Pankov, I. V.; Nikolskiy, A. V.; Kozakov, A.T.; Moguchikh, E.A.; Alekseenko, A.A. Influence of the Heat Treatment on Structural and Functional Characteristics of the PtCu/C Electrocatalysts on Various Carbon Supports. Journal of Solid State Electrochemistry 2024, 28, 3367–3381, doi:10.1007/S10008-024-05920-8.

2. Paperzh, K.O.; Alekseenko, A.A.; Volochaev, V.A.; Pankov, I. V.; Safronenko, O.A.; Guterman, V.E. Stability and Activity of Platinum Nanoparticles in the Oxygen Electroreduction Reaction: Is Size or Uniformity of Primary Importance? Beilstein Journal of Nanotechnology 12:49 2021, 12, 593–606, doi:10.3762/BJNANO.12.49.

3. Moguchikh, E.A.; Alekseenko, A.A.; Pankov, I. V.; Alekseenko, D. V.; Guterman, V.E. Changes in the Microstructure and Electrochemical Behavior of Pt/C Electrocatalysts under Various Stress Testing Conditions. Nanobiotechnology Reports 2023, 18, S301–S315, doi:10.1134/S2635167624600081.

4. Nagai, T.; Jahn, C.; Jia, H. Improved Accelerated Stress Tests for ORR Catalysts Using a Rotating Disk Electrode. J Electrochem Soc 2019, 166, F3111–F3115, doi:10.1149/2.0161907JES.

5. Moguchikh, E.A.; Paperzh, K.O.; Alekseenko, A.A.; Gribov, E.N.; Tabachkova, N.Y.; Maltseva, N. V.; Tkachev, A.G.; Neskoromnaya, E.A.; Melezhik, A. V.; Butova, V. V.; et al. Platinum Nanoparticles Supported on Nitrogen-Doped Carbons as Electrocatalysts for Oxygen Reduction Reaction. J Appl Electrochem 2022, 52, 231–246, doi:10.1007/S10800-021-01629-Y.

6. Wei, P.J.; Yu, G.Q.; Naruta, Y.; Liu, J.G. Covalent Grafting of Carbon Nanotubes with a Biomimetic Heme Model Compound To Enhance Oxygen Reduction Reactions. Angewandte Chemie International Edition 2014, 53, 6659–6663, doi:10.1002/ANIE.201403133.

7. Khodabakhshi, S.; Fulvio, P.F.; Andreoli, E. Carbon Black Reborn: Structure and Chemistry for Renewable Energy Harnessing. Carbon N Y 2020, 162, 604–649, doi:10.1016/J.CARBON.2020.02.058.
